# Identification and validation of a hypoxia-immune signature for overall survival prediction in lung adenocarcinoma

**DOI:** 10.3389/fgene.2022.975279

**Published:** 2022-10-03

**Authors:** Yong Li, Huiqin Huang, Meichen Jiang, Nanding Yu, Xiangli Ye, Zhenghui Huang, Limin Chen

**Affiliations:** ^1^ Department of Respiration Medicine, Fujian Medical University Union Hospital, Fuzhou, China; ^2^ Fujian Provincial Key Laboratory of Medical Testing, Fujian Academy of Medical Sciences, Fuzhou, China; ^3^ Department of Pathology, Fujian Medical University Union Hospital, Fuzhou, China

**Keywords:** LUAD, hypoxia, immune, tumor microenvironment, signature

## Abstract

**Objective:** The interaction between immunity and hypoxia in tumor microenvironment (TME) has clinical significance, and this study aims to explore immune-hypoxia related biomarkers in LUAD to guide accurate prognosis of patients.

**Methods:** The LUAD gene expression dataset was downloaded from GEO and TCGA databases. The immune-related genes and hypoxia-related genes were acquired from ImmPort and MSigDB databases, respectively. Genes related to immune and hypoxia in LUAD were obtained by intersection. The significantly prognostic genes in LUAD were obtained by LASSO and Cox regression analyses and a prognostic model was constructed. Kaplan-Meier and receiver operating characteristic curves were generated to evaluate and validate model reliability. Single-sample gene set enrichment analysis (ssGSEA) and gene set variation analysis (GSVA) were employed to analyze immune cell infiltration and pathway differences between high- and low-risk groups. Nomogram and calibration curves for survival curve and clinical features were drawn to measure prognostic value of the model.

**Results:** The prognosis model of LUAD was constructed based on seven immune-hypoxia related genes: S100P, S100A16, PGK1, TNFSF11, ARRB1, NCR3, and TSLP. Survival analysis revealed a poor prognosis in high-risk group. ssGSEA result suggested that activities of immune cells in high-risk group was remarkably lower than in low-risk group, and GSVA result showed that immune-related pathway was notably activated in low-risk group.

**Conclusion:** Immune-hypoxia related genes were found to be prognostic biomarkers for LUAD patients, based on which a 7-immune-hypoxia related gene-signature was constructed. This model can assess immune status of LUAD patients, and provide clinical reference for individualized prognosis, treatment and follow-up of LUAD patients.

## Introduction

Lung cancer is the most frequent fatal disease. According to statistics, 2.09 million new diagnoses and 1.76 million deaths occurred in 2020, and morbidity and mortality are on the rise (Bade and Dela Cruz, 2020). As a histology of non-small cell lung cancer (NSCLC), lung adenocarcinoma (LUAD) with high morbidity and mortality is featured by highly invasive and disruptive growth characteristics (Li et al., 2014). Occurrence, development, recurrence, and patient’s prognosis of tumors are associated with pathological type, clinical stage and tumor gene expression (Müller et al., 2016). With the constant development of biotechnology and the rapid development of precision medicine, LUAD-targeted drug research and treatment methods are becoming increasingly mature. Biomarkers identified include EGFR, TP53, AKT1, KRAS and PTEN (Bean et al., 2007; Bleeker et al., 2008; Jin et al., 2010). TP53 mutation is a prevalent mutated gene in LUAD patients and attenuates the immune response of early LUAD patients (Zhu et al., 2021). It is a prognostic biomarker for LUAD patients (Sun et al., 2020). Additionally, phosphorylated AKT1 and MAPK3/1 can co-activate RELA, and they can also activate NF-κB through miR-3613-5p, thus modulating LUAD cell proliferation (He et al., 2020). Currently, these biomarkers have been applied in surgery, targeted therapy, radiotherapy, immunotherapy and chemotherapy. Although the therapeutic effect is evident, only a few patients can benefit from it. In addition, due to local recurrence and remote metastasis, LUAD patients have a 5-year survival rate of 4%–17% (Bade and Dela Cruz, 2020). Thus, to further explore prognostic biomarkers involved in the progression of LUAD is of great significance.

The occurrence and progression of tumor is accompanied by hypoxia, which is one of the markers of TME, and has close relation with the excessive growth, distal metastasis, recurrence and drug resistance of many solid tumors (Rey et al., 2017). Current studies believe that tumor cells under hypoxia can increase expression of hypoxia inducible factor-1 (HIF-1), while overexpressed HIF can be a transcription regulatory factor on target genes. Under the regulation of the transcription level of target genes, many biological processes, including tumor angiogenesis, autophagy, apoptosis, immune regulation, energy metabolism and pH regulation, are affected, which promotes tumor cells to adapt to the hypoxia environment, so that the continuous survival, proliferation and invasion of tumor cells are achieved (Weidemann and Johnson, 2008; Majmundar et al., 2010; Semenza, 2010). Meanwhile, the connection between tumor hypoxia environment and tumor immunity is very close. It has been documented that T cells exhibit immune dysfunction in anoxic microenvironment (Cubillos-Zapata et al., 2017). Specifically, tumors in anoxic environment can induce the expression of CCL28 to recruit a large number of Tregs, thus promoting tumor angiogenesis and tolerance (Facciabene et al., 2011). It is noteworthy that tumor-associated macrophages (TAM) are important components of hypoxic TME, and TAM enhances its inhibitory effect on T cells by expressing HIF-1α (Doedens et al., 2010). Currently, clinical trials have demonstrated that targeting HIF-1α can enhance the viability of immune cells (Sun et al., 2001). These studies suggest that targeting hypoxia pathway can improve tumor immunotherapy, and targeted detection of hypoxia may present patients the benefit of immunotherapy. In conclusion, these findings highlight potential of hypoxia as a prognostic marker for LUAD, and the identification of LUAD prognostic markers from the perspective of hypoxic TME is an important entry point.

In this study, on the basis of gene expression profile of LUAD patients in TCGA database, seven prognostic markers of immune-hypoxia related genes were identified, and a prognostic model was established. TME immune cell infiltration status of patients with varying risk ratings was evaluated to further elucidate the influence of hypoxia environment and immune infiltration on prognoses of LUAD patients**,** improve understanding of LUAD hypoxic TME, and lay scientific basis for the subsequent study on tumorigenesis, development of LUAD and new targeted therapy.

## Materials and methods

### Data acquisition

The mRNA expression dataset TCGA-LUAD (normal: 59, tumor: 535), as well as the corresponding clinical information data, were accessed from TCGA database (https://portal.gdc.cancer.gov/). GSE31210 (normal: 20, tumor: 226, platform GPL570) and GSE72094 (tumor: 446, platform GPL15048) were downloaded from GEO database (https://www.ncbi.nlm.nih.gov/geo/). Samples with survival time less than 30 days in each data set were excluded. Finally, 460, 226 and 386 cancer samples in TCGA-LUAD, GSE31210 and GSE72094 were reserved, respectively.

### Identification of differentially expressed hypoxia-immune related genes

1811 immune-related genes (Supplementary Table S1) and 200 hypoxia-related genes (Supplementary Table S2) were acquired from ImmPort database (https://www.immport.org/home) and Molecular Signature Database (MSigDB) (https://www.gsea-msigdb.org/gsea/msigdb/), wherein 652 genes were not identified in TCGA-LUAD, GSE31210 and GSE72094 data sets. Therefore, survival analysis was conducted after removing these 652 genes from the data set.

### Construction and assessment of a hypoxia-immune related signature

Based on survival information of LUAD patients, PACKAGE survival (Therneau and Grambsch, 2000) was applied to perform univariate Cox regression analysis on immune-hypoxia related genes in TCGA-LUAD and GSE72094 cohorts, and genes significantly correlated with patient overall survival (OS) were screened (*p* < 0.01). Intersection of survival-related genes in the two cohorts was taken, and overlapping genes were used for further analysis. Next, TCGA-LUAD was taken as the training set. By using R package glmnet, LASSO Cox regression analysis was done on genes that were remarkably associated with OS (Friedman et al., 2010). Appropriate punishment parameter lambda was selected by the ten-fold cross-validation selection method to remove genes with strong correlation to reduce model complexity. Next, by using R package survival, multivariate Cox regression analysis was conducted on genes screened by LASSO Cox analysis to obtain prognostic genes with immune-hypoxia feature in LUAD. The prognostic risk assessment model of LUAD was built according to risk coefficient and expression of feature genes.

$$Riskscore=\overset{n}{\underset{i=1}{\sum }}\exp_{i}*\beta_{i}$$

*n* is the number of selected genes, *exp*_*i*_ represents expression of gene *i*, and *β*_*i*_ indicates regression coefficient of gene *i*.

The riskscore of each cancer patient in TCGA-LUAD cohort was calculated, and patients were assigned into high- or low-risk groups by the median riskscore value. Survival analysis was conducted, and R package survminer was employed to generate survival curves of the two risk groups (https://cran.r-project.org/web/packages/survminer/index.html). The relevant survival state graph was plotted according to the riskcsore of the samples. Subsequently, ROC curves for prognostic models were plotted by R package survival ROC to predict patients’ OS at 1, 3 and 5 years (https://rdrr.io/cran/survivalROC/man/survivalROC.html). Independent data sets GSE31210 and GSE72094 were used as validation sets to verify accuracy of the model. Finally, effects of prognosis-related gene expression on prognoses of LUAD patients were analyzed on the GEPIA website (http://gepia.cancer-pku.cn/index.html).

### Single-sample gene set enrichment analysis

Twenty-nine kinds of immune cells and immune-related functional gene sets were got from the published literature (Jiang et al., 2021). We assessed immune cell infiltration level in TME of LUAD patients based on these 29 immune data sets. ssGSEA was on samples in two groups using R package GSVA (Hänzelmann et al., 2013) to study immune cell infiltration level of patients with different riskscores.

### Gene set variation analysis

GSVA estimates pathway activation variation in sample populations in an unsupervised manner, which is an excellent molecular mapping method (Hänzelmann et al., 2013). Enrichment scores for varying biological pathways in each sample in TCGA-LUAD cohort were calculated using R package GSVA. From MsigDB (http://software.broadinstitute.org/gsea/msigdb/index.jsp), the “c2.cp.kegg.v7.0.symbols.gmt” data were downloaded as reference. Through GSVA analysis, the differential pathways between groups were obtained. The differential enrichment pathways were further identified, with the standard of FDR < 0.01 and *p* < 0.01.

### Riskcore independence verification and nomogram construction

Univariate and multivariate Cox regression analyses were conducted using riskscore and clinical information (gender, age, T, N, stage) to verify whether riskscore could be an independent prognostic factor for LUAD. To predict 1-, 3-, and 5-year survival rates of LUAD patients, a nomogram was constructed based on riskscore and clinicopathological information using R package rms (Huang et al., 2019). Finally, calibration curves were drawn to evaluate consistency in predicted survival time and actual survival time of patients.

### Statistics

All statistical analyses were done on R software (3.3.1). The results were not statistically significant if data did not follow a normal distribution. Differences between groups were compared by Kruskal–Wallis test or Wilcoxon rank-sum test. Cox Regression model was utilized to perform univariate and multivariate analyses. Survival differences were assessed by logarithmic rank test. When *p* value < 0.01, the data were statistically significant.

## Results

### Identification of immune-hypoxia-related prognostic genes

**FIGURE 1:**
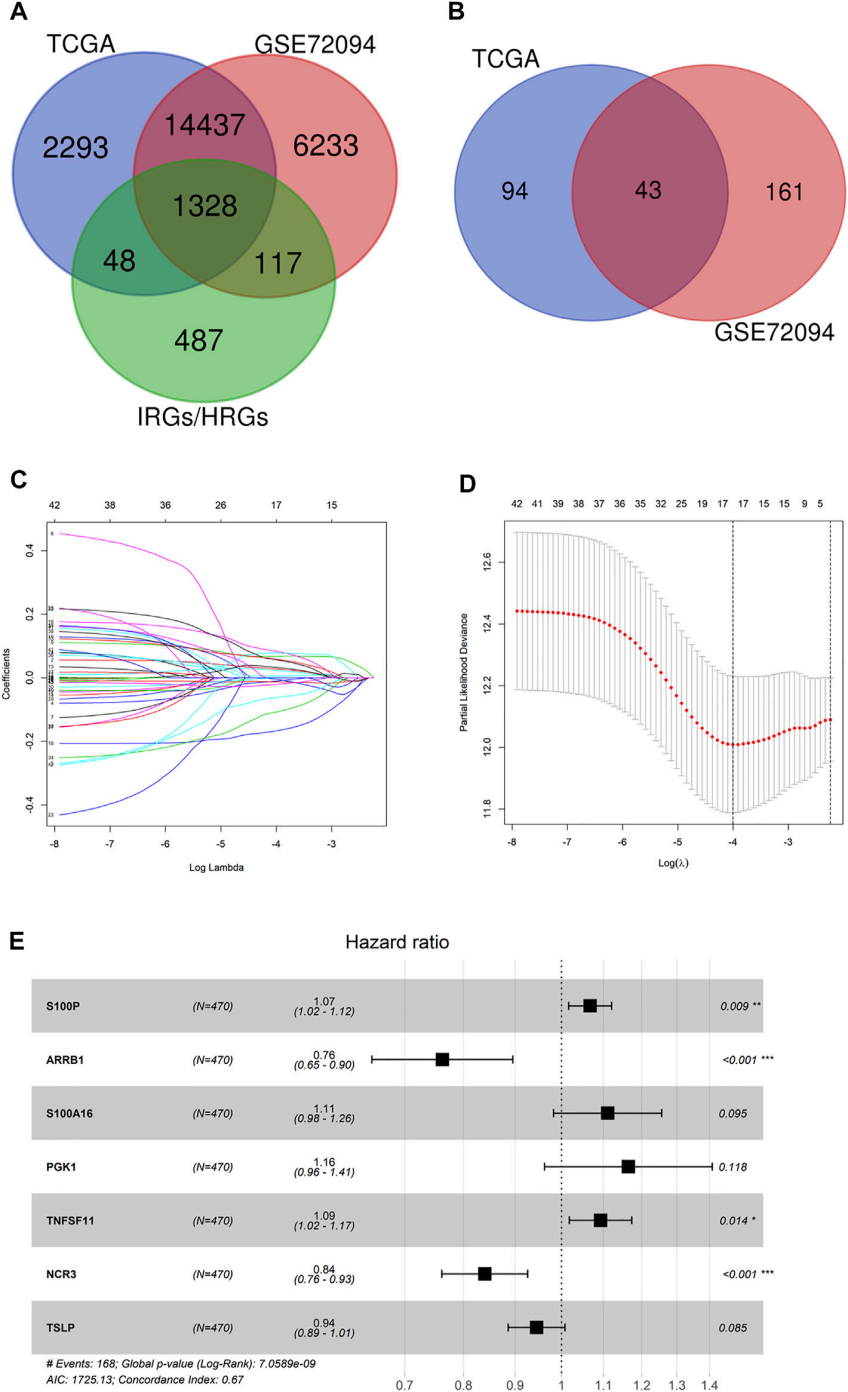
Identification of immune-hypoxia related prognostic genes in LUAD. **(A)** Venn diagram of immune-hypoxia-related genes in TCGA-LUAD, GSE72094 and IRGs/HRGs; **(B)** Venn diagram of OS-related genes in TCGA-LUAD and GSE72094; **(C)** Gene coefficient spectrum in LASSO Cox regression analysis of 43 OS-related genes in TCGA-LUAD cohort; **(D)** Selection of optimum penalty parameter (λ) in LASSO Cox regression model; **(E)** Forest plot of Cox regression analysis, *p < 0.05, **p < 0.01, ***p < 0.001.

1,328 immune-hypoxia-related genes that were overlapped in TCGA-LUAD and GSE72094 were identified (Figure 1A). Based on these genes as well as the survival information of TCGA-LUAD and GSE72094 cohorts, univariate Cox regression analysis was completed, and 137 and 204 genes associated with survival in the two cohorts were obtained, respectively. By intersecting, 43 genes related to patient survival were identified (Figure 1B). LASSO Cox regression evaluated the regression coefficients of 43 genes associated with survival, and a fitting curve was plotted (Figure 1C). Then, an appropriate punishment parameter lambda was selected according to 10-fold cross-validation. Results showed that when lambda was −4, the model contained 17 survival-related genes, which had the lowest complexity (Figure 1D). The above 17 genes underwent multivariate Cox regression analysis, and complexity and excellence of different models were evaluated according to Akaike Information Criterion (AIC). The model showing the lowest AIC value was chosen as the prognostic model, which contained seven immune-hypoxia-related genes significantly related to prognoses of LUAD patients. Among these genes, S100P, S100A16, PGK1 and TNFSF11 were risk factors and ARRB1, NCR3 and TSLP were protective factors (Figure 1E). As the risk coefficients and expression of seven genes mentioned above, the immune-hypoxia feature prognostic model of LUAD was riskscore = 0.065145908*coef (S100P) − 0.27104694*coef (ARRB1) + 0.104952032*coef (S100A16) + 0.152008632*coef (PGK1) + 0.088969471*coef (TNFSF11) − 0.174725763*coef (NCR3) − 0.056725904 *coef (TSLP). In conclusion, seven immune-hypoxia-related genes in LUAD were identified by LASSO and Cox repression analyses combined with patient survival information.

### Performance assessment of the prognostic model

**FIGURE 2:**
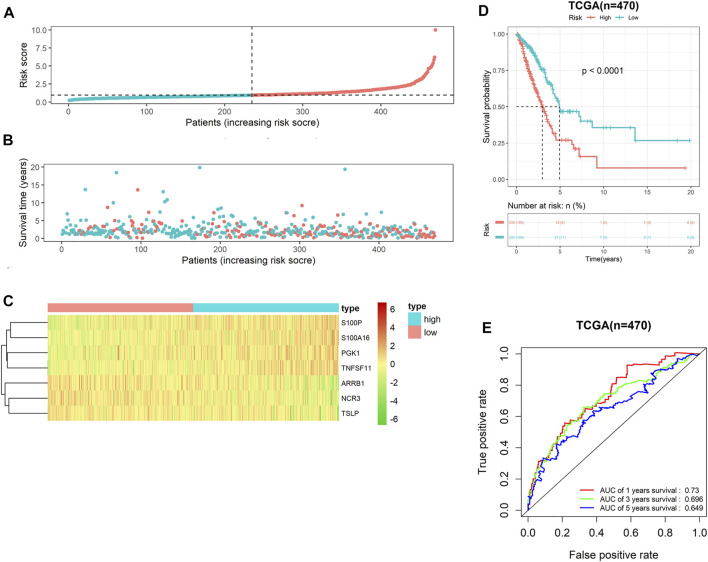
Performance assessment of the 7-gene signature in TCGA-LUAD cohort **(A)** Riskscore arrangement of LUAD patients; **(B)** Riskscore chart of patients in high- and low-risk groups; **(C)** Distribution of survival status of patients in two risk groups; **(D)** Survival curves of patients in two risk groups; **(E)** ROC curves of 1-, 3- and 5-year survival of LUAD patients predicted by the 7-gene signature.

Following risk coefficient and expression of 7-gene signature, the riskscore of each LUAD patient in TCGA-LUAD cohort was computed, and riskscore distribution map was plotted. Patients were assigned into either high- or low-risk groups (Figure 2A). On the basis of survival time and riskscore of LUAD patients in TCGA-LUAD cohort, we drew a scatter plot of patients’ survival time, and the results displayed a negative correlation of survival time and riskscore (Figure 2B). Meanwhile, we plotted the expression heat maps of the seven feature genes in two risk groups. The heat maps showed that risk factors S100P, S100A16, PGK1 and TNFSF11 expressed highly in high-risk groups, while protective factors ARRB1, NCR3 and TSLP expressed highly in low-risk group (Figure 2C). Survival curve showed that prognoses of patients in low-risk group was notably better than in high-risk group (Figure 2D). Subsequently, ROC curves of 1-, 3- and 5-year survival predicted by the 7-gene signature were plotted. AUC values of 1-, 3- and 5- survival were 0.73, 0.696 and 0.649, respectively (Figure 2E), suggesting that this prognostic model could evaluate the prognoses of LUAD patients well. Overall, the 7-gene signature presented favorable predictive performance in the training set TCGA-LUAD cohort.

**FIGURE 3:**
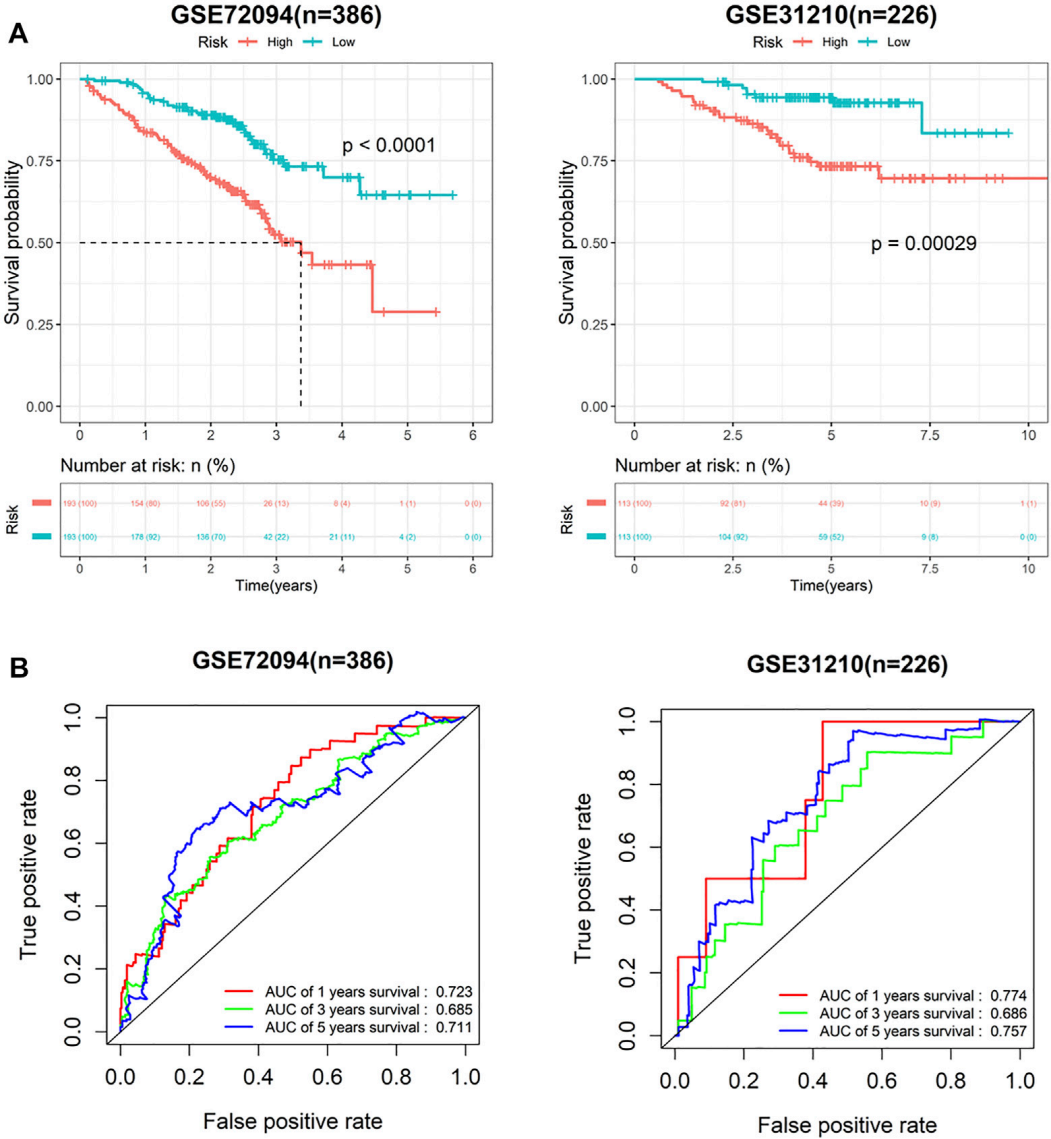
Performance verification of 7-gene signature in GEO cohort. **(A)** Survival curves of high- and low-risk groups in GSE72094 and GSE31210 cohort; **(B)** ROC curves of 1-, 3- and 5-year survival predicted by 7-gene signature in GSE72094 and GSE31210 cohorts.

**FIGURE 4:**
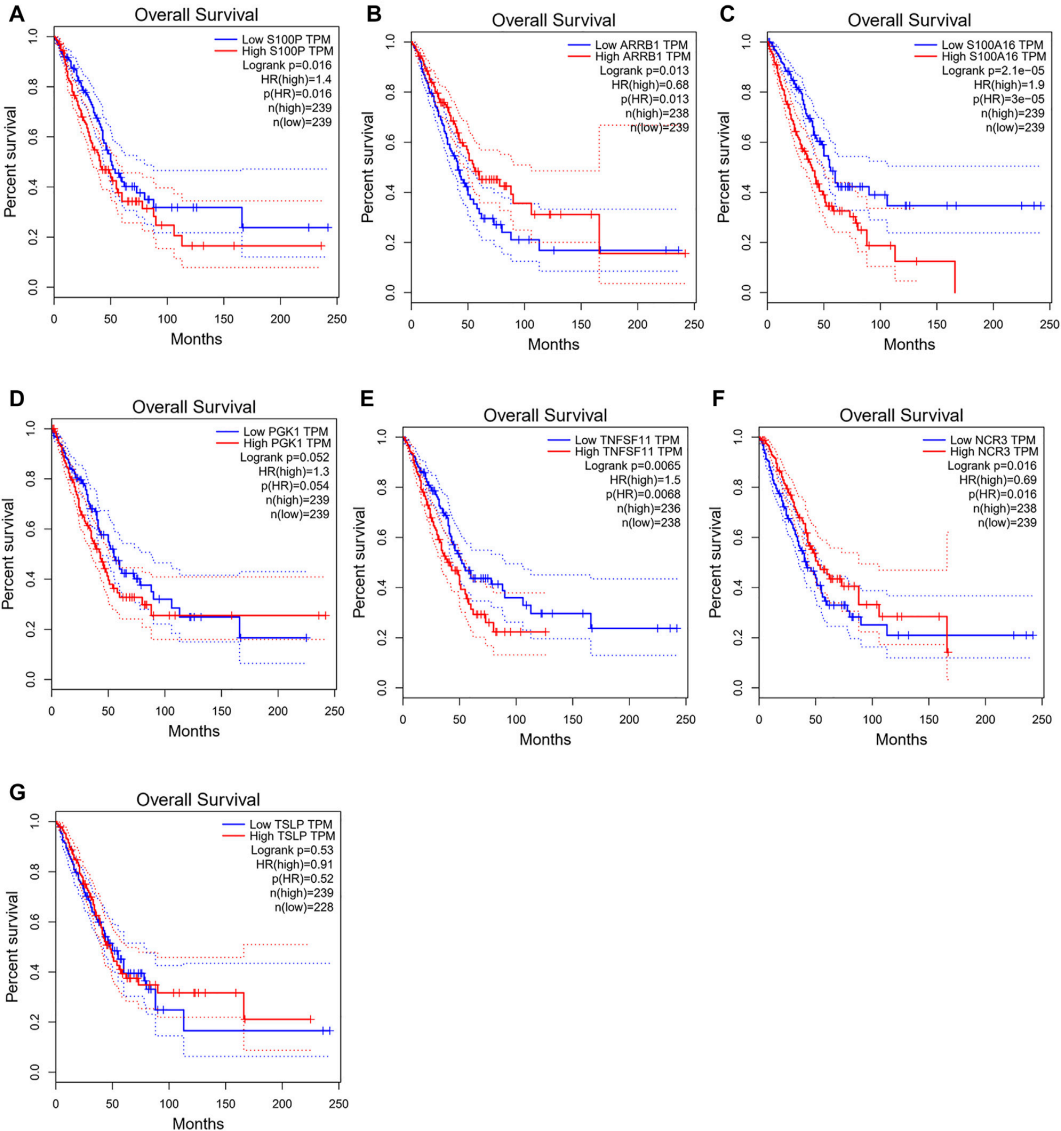
Influences of the seven genes on prognoses of LUAD patients. **(A–G)** Survival curves of patients with high and low expression of S100P **(A)**, ARRB1 **(B)**, S100A16 **(C)**, PGK1 **(D)**, TNFSF11**(E)**, NCR3 **(F)** and TSLP**(G)**.

To verify predictive potential of the 7-gene signature, riskscore of patients in GSE72094 and GSE31210 data sets was calculated, and samples were assigned into high- or low-risk groups. Survival analysis results showed poor OS in high-risk group (Figure 3A). ROC curves of 1-, 3- and 5-year survival predicted by signature in GSE72094 and GSE31210 data sets were plotted. AUC values of 1-, 3- and 5-year survival in GSE72094 were 0.723, 0.685 and 0.711, respectively, while in GSE31210 were 0.774, 0.686 and 0.757 (Figure 3B). In addition, to explore the influence of seven genes on prognoses of LUAD patients, the survival curve was plotted. The results showed that S100P, TNFSF11, ARRB1 and NCR3 directly affected the prognoses of LUAD patients, while S100A16, PGK1 and TSLP did not (Figures 4A–G). In conclusion, this 7-gene signature based on immune-hypoxia related genes was able to accurately predict prognoses of LUAD patients.

### Single-sample gene set enrichment analysis of high- and low-risk groups

**FIGURE 5:**
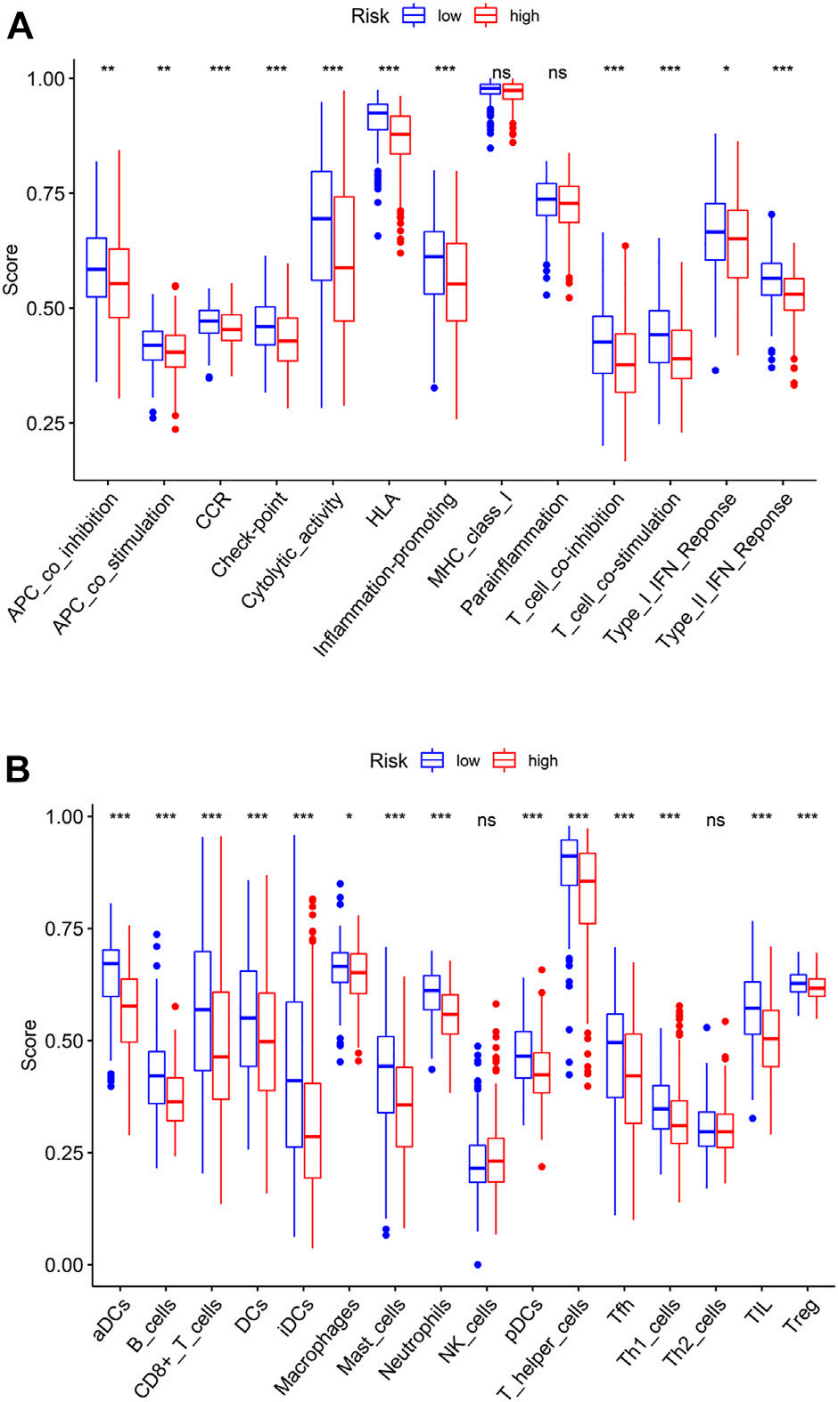
ssGSEA in high- and low-risk groups. **(A)** Immune-related gene sets in high- and low-risk groups; **(B)** Boxplot of immune cell differences in the two risk groups.

To evaluate the differences in TME immune cell infiltration between high- and low-risk patients, ssGSEA was done. Results exhibited that the expression of immunoreactive gene sets, including APC_co_stimulation, APC_co_inhibition, CCR, Check-point, HLA, Cytolytic_activity, Parainflammation, Inflammation-promoting, T_cell_co-stimulation, T_cell_co-inhibition, Type_II_IFN_Response and Type_I_IFN_Response in low-risk group were prominently higher than those in high-risk group (Figure 5A), and the infiltration levels of immune cells B_cells, aDCs, DCs, iDCs, CD8+_T_cells, Mast_cells, Macrophages, Neutrophils, pDC, Tfh, T_helper_cells, TIL, Th1_cells and Treg were higher (Figure 5B). Based on the above findings, we noted that the immune score of patients in high-risk group was relatively low. Combined with the results of patient survival analysis, we concluded that patients in high-risk group may be immunosuppressed.

### Gene set variation analysis results of high- and low-risk groups

**FIGURE 6:**
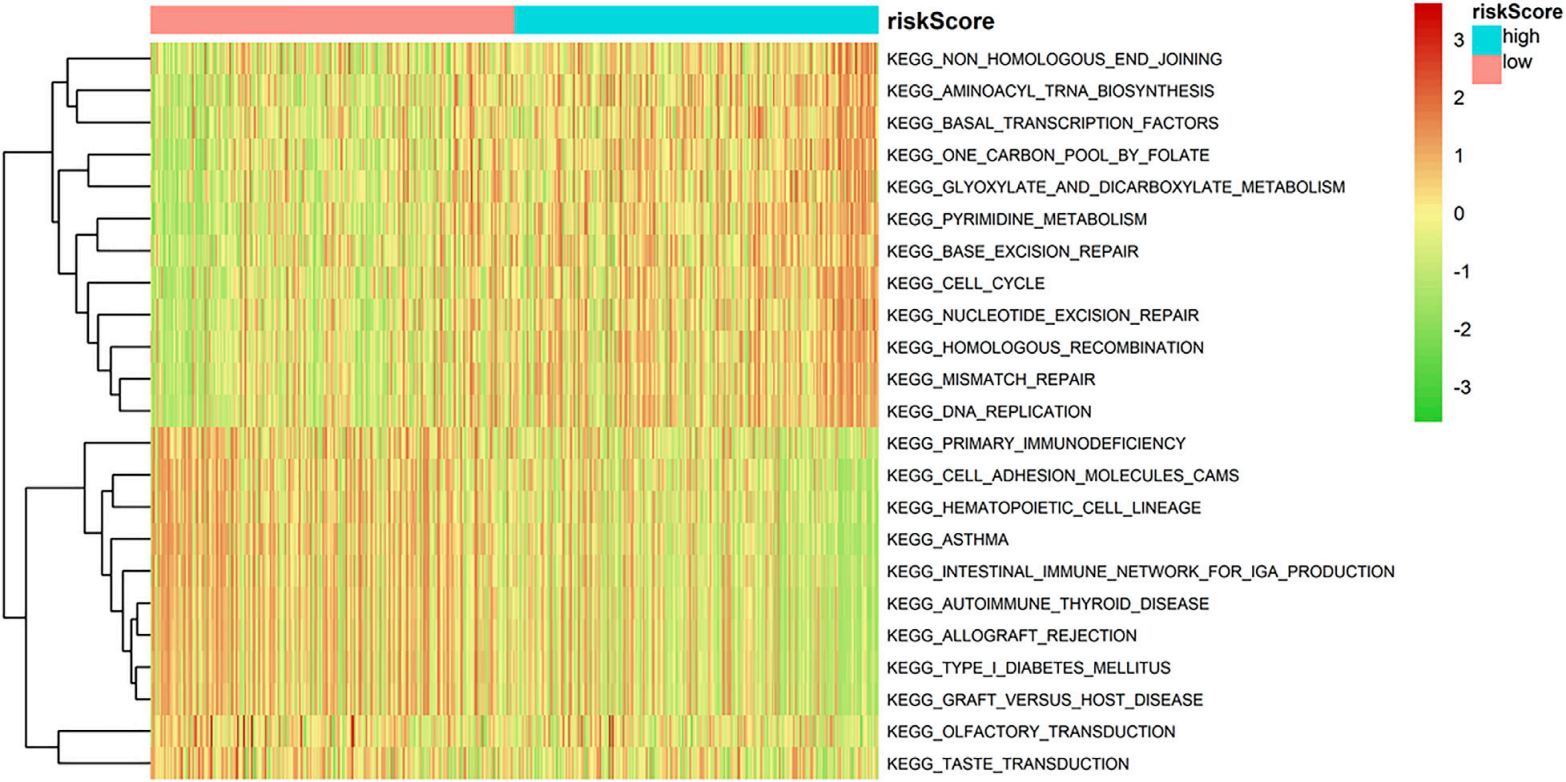
GSVA in high- and low-risk groups.

Based on enrichment score of each sample, we determined differences in enrichment pathways between two risk groups by GSVA (FDR < 0.01, *p* < 0.01). Heat map indicated that the pathways related to metabolism and mismatch repair, inluding KEGG_ONE_CARBON_POOL_BY_FOLATE, KEGG_GLYOXYLATE_AND _DICARBOXYLATE_METABOLISM, KEGG_PYRIMIDINE_METABOLISM, KEGG_HOMOLOGOUS_RECOMBINATION, KEGG_MISMATCH_REPAIR and KEGG_DNA_REPLICATION, were substantially activated in high-risk group. In low-risk group, immune-related pathways, including KEGG_INTESTINAL_IMMUNE _NETWORK_FOR_IGA_PRODUCTION, KEGG_PRIMARY_IMMUNODEFICIENCY, KEGG_ALLOGRAFT_REJECTION, KEGG_AUTOIMMUNE_THYROID_DISEASE and KEGG_GRAFT_VERSUS_ HOST_DISEASE, were significantly activated (Figure 6). Based on these results, patients in high-risk group may be immunosuppressed.

### Validation of riskscore independence

**FIGURE 7:**
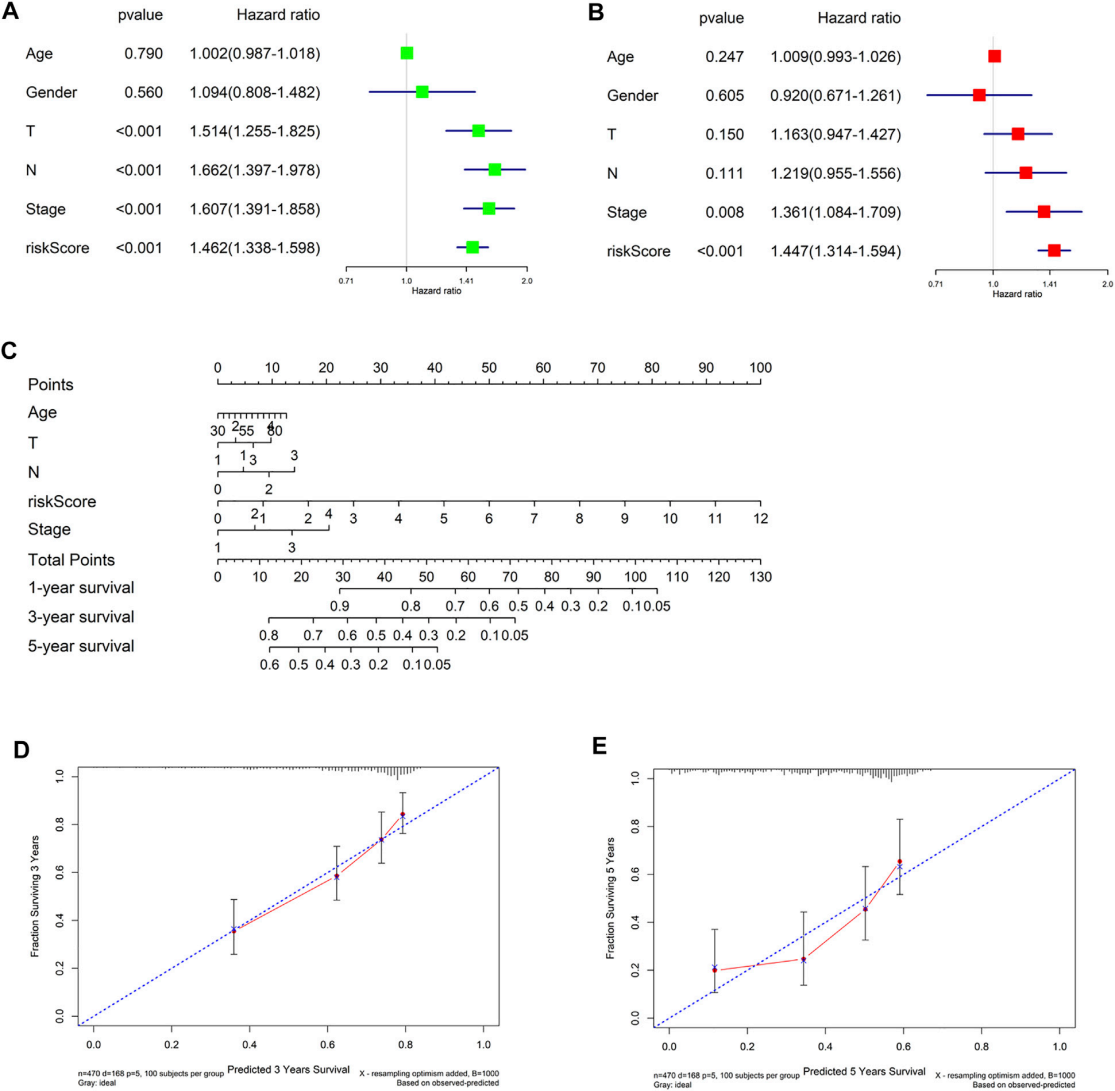
Construction and evaluation of the nomogram. **(A,B)** Univariate and multivariate Cox regression analyses for riskscore and clinicopathological features; **(C)** The nomogram constructed from riskscore and clinicopathological features was used to predict 1-, 3-, and 5-year survival of patients in the training cohort; **(D,E)** The calibration curve was used to describe the consistency between nomogram prediction of 3- and 5-year survival and actual survival.

Finally, in TCGA-LUAD data set, univariate and multivariate Cox regression analyses were conducted combining clinical information (gender, age, T, N and Stage) with riskscore. The forest plots suggested that riskscore could be an independent factor of LUAD OS (Figures 7A,B). Then we integrated the clinical information to generate a nomogram to predict 1-, 3-, and 5-year OS of LUAD patients (Figure 7C). Finally, nomogram calibration curves presented that the predicted and actual OS values of LUAD patients were highly consistent (Figures 7D,E). In conclusion, the 7-gene signature could effectively predict prognoses of LUAD patients.

## Discussion

It has been confirmed that immune and hypoxic microenvironment are key factors in tumorigenesis and progression. Immunosuppression induced by hypoxia microenvironment is the key factor of tumor immunosuppression (Chen and Mellman, 2013; Kim and Chen, 2016). Therefore, based on hypoxia and immune-related genes, we explored LUAD hypoxia-immune microenvironment and established the LUAD prognosis model with 7-immune-hypoxia related genes, which presented favorable performance in predicting prognoses.

Herein, seven prognostic biomarkers were identified by Cox regression analysis of immune-hypoxia related genes in LUAD. There were 4 risk factors including S100P, S100A16, PGK1 and TNFSF11, and three protective factors including ARRB1, NCR3 and TSLP. S100P is highly expressed in many cancer tissues, including triple negative breast cancer, and it is related to adverse clinical outcomes such as lymphatic metastasis and tumor growth (Kikuchi et al., 2019). This is consistent with the prognostic trend of S100P on LUAD in this study. Li et al. (2016) manifested that in gallbladder cancer, LASP-1 can arrest cell cycle in G2/M phase by regulating S100P. S100A16 (Fang et al., 2021) and ARRB1 (Zhang et al., 2017) are also related to cell cycle, In this study, high- and low-risk groups differed in degree of enrichment of cell cycle-related pathways. Tumor metastasis is one of the reasons that make cancer uncurable. The occurrence of EMT is accompanied by the occurrence of cancer and reduces cell adhesion and enhances cell migration (Laubli and Borsig, 2019). TNFSF11, also known as RANKL, has been shown by Mineon Park et al. (2021) to substantially improve PC3 cell migration and invasion and enhance EMT after treatment of PC3 cells with RANKL. Meanwhile, ARRB1, S100P, S100A16, and TSLP that are in our established model are also associated with cancer metastasis (Barooei et al., 2015; Zhu et al., 2016; Du et al., 2020; Xu et al., 2020). This is in line with our study that high- and low-risk groups differ in degree of enrichment of cell adhesion-related signaling pathways in GSVA analysis. NCR3 expression is an immune parameter of immune cells in advanced NSCLC (Charrier et al., 2019). The RANK/RANKL signaling in M2 macrophages regulates chemokines production and promotes Treg lymphocytes proliferation, being conducive to immunosuppressive environment (Fujimura et al., 2015). This is also in congruous with our results that the high-and low-risk groups differ in degree of enrichment with immune-related diseases, indicating that the signature genes are related to immunity. Hence, these signature genes are associated with the malignant progression of LUAD and can be applied as prognostic markers for LUAD. Hence, it is reasonable to use the prognostic model constructed by these genes for predicting prognostic risk of LUAD patients.

Immune system is the “scavenger” in the body. In cancer patients, impaired cancer immune circulation leads to anti-tumor immune deficiency, which is also the cause of cancer occurrence and development (Starzer et al., 2022). We found that ssGSEA scores of HLA, CD8+ T cells, T helper cells and B cells in low-risk group were noticeably higher than in high-risk group. These cytokines and immune cells play important roles in tumor immunity. For example, HLA can present endogenous antigens and activate CD8+ T cells. CD8+ T cells recognizes cancer cells or infected cells, and also activate B cells to produce different types of antibodies to exert the immune function (Rock et al., 2016). T helper cells have rich classifications, in which Tfh cells can help B cells produce antibodies by producing IL-21 and expressing Bcl6. Treg cells can modulate the immune response to maintain immune cell homeostasis (Zhu and Zhu, 2020). In the present study, patients in low-risk group had substantially better prognoses than in the other group, which may be caused by activation of CD8+ T cells in TME by T helper cells and secretion of large amounts of cytokines by B cells. The TME of patients in the low-risk group was in immune activation.

In summary, seven Immune-hypoxia related genes were screened out in LUAD by bioinformatics methods in this study, and a LUAD prognostic assessment model was constructed. Our results showed that this model had good accuracy and universality, and were able to predict clinical outcome of LUAD patients and provide treatment reference. However, deficiencies still exist. Firstly, the data for constructing and verifying the 7-gene model were all retrospective data from TCGA and GEO databases, which may lead to selection bias. In that case, we need to include more prospective data in subsequent studies to further confirm the clinical efficacy of the model and establish our own database for prospective cohort testing, so as to increase the reliability of prognostic signature genes. Secondly, the specific mechanism of 7-gene in LUAD is still unclear, and further experimental exploration is needed. Thirdly, few studies have demonstrated the relationship between NCR3 and LUAD, and thus, studies are warranted to clarify its mechanism. At the same time, we analyzed differential pathways between two LUAD risk groups, supplying a foundation for further exploring interaction mechanism between LUAD hypoxia and immune infiltration.

## Data Availability

The original contributions presented in the study are included in the article/Supplementary Material, further inquiries can be directed to the corresponding author.
